# Improving Documentation of Driving Status and DVLA Advice in Liaison Psychiatry: A Quality Improvement Project

**DOI:** 10.1192/bjo.2026.11263

**Published:** 2026-06-30

**Authors:** Marwa Ali

**Affiliations:** West London NHS, London, United Kingdom

## Abstract

**Aims:**

Patients assessed by liaison psychiatry frequently have mental health conditions or are prescribed medications that may impair fitness to drive. General Medical Council (GMC) and Driver and Vehicle Licensing Agency (DVLA) guidance require clinicians to assess driving status, provide appropriate advice, and document this clearly. Local observations suggested that this aspect of assessment was frequently omitted, posing patient safety and medico-legal risks.

Aims were to improve the identification, documentation, and communication of driving status and DVLA advice in liaison psychiatry assessments within an acute hospital setting.

**Methods:**

A baseline audit of 20 consecutive liaison psychiatry assessments was conducted to assess documentation of driving status and DVLA advice when indicated. A series of Plan–Do–Study–Act (PDSA) cycles were implemented. Interventions included the addition of a “Driving Status and DVLA Advice” prompt to the assessment template, regular verbal and email reminders to the multidisciplinary team, sharing real-case learning points, and displaying visual prompts within the team office. Weekly re-audits were undertaken to assess change over time.

**Results:**

At baseline, driving status was documented in 5% of assessments, and no patients received documented DVLA advice when indicated. Following sequential PDSA cycles, documentation of driving status improved progressively to 50% by week three. Provision of DVLA advice also increased, reaching 13.6% of assessments by week three. Improvements were most marked following repeated reinforcement strategies alongside system-level prompts. Persistent gaps were identified in consistently providing DVLA advice once drivingstatus was confirmed.

**Conclusion:**

Simple, low-cost system and behavioural interventions led to meaningful improvements in documenting driving status and DVLA advice in liaison psychiatry assessments. Sustained improvement required repeated reinforcement and team engagement. Embedding prompts into assessment templates and incorporating DVLA guidance into staff induction are key to long-term sustainability.

